# The relationship between perceptions toward advertising and consumption of energy-dense nutrient-poor foods among adults in the United States: results from a national survey

**DOI:** 10.3389/fpubh.2025.1516164

**Published:** 2025-02-05

**Authors:** Chan L. Thai, Jacqui Villarreal, Jacqueline A. Thai

**Affiliations:** Department of Communication, Santa Clara University, Santa Clara, CA, United States

**Keywords:** obesity prevention, food advertising, trust in advertising, health communication, junk food marketing, national study, media literacy interventions

## Abstract

**Introduction:**

Much of the research on the effects of food advertising has been focused on children and adolescents; however, adults may also be influenced. Prior research has also shown that exposure to food advertisements have impacted the consumption behaviors of adults. The purpose of this study is to explore (1) the differences in perceptions toward and trust in food advertisements between racial/ethnic population subgroups; and (2) the associations between perceptions toward food advertising and the consumption of energy-dense, nutrient poor (EDNP) foods among adults using data from a national data set (*n* = 1,535).

**Methods:**

Data from the National Cancer Institute’s Family Life, Activity, Sun, Health, and Eating (FLASHE) survey were utilized in this secondary data analysis study. We conducted one-way ANOVAs to evaluate demographic subgroup differences within advertising trust and perceptions, and utilized bivariate and multivariable linear regression models to examine associations between (1) the perceptions toward and trust in food advertisements between racial/ethnic population subgroups; and (2) the associations between perceptions toward food advertising and the consumption of EDNP foods, while controlling for sociodemographic factors.

**Results:**

Results show significant differences between racial/ethnic groups on advertising perceptions (*F* = 8.59, *p* < 0.0001). Planned contrasts show that there was a statistically significant and meaningful difference (*p* = 0.04) between Non-Hispanic Blacks (mean = 2.85) and Non-Hispanic Whites (mean = 2.52) for trust in food advertising. Regression analyses show that as positive perceptions toward food advertising increase among adults, there is an increase in daily frequency of consumption of EDNP foods and drinks (𝛽 = 0.15, *p* < 0.0001). This pattern was similar for trust in food advertising (𝛽 = 0.13, *p* < 0.0001).

**Discussion:**

Given that previous studies have shown that ethnic minority groups, particularly Non-Hispanic Blacks, are more likely to be exposed to unhealthy food advertisements across various types of media, such as TV, billboards, and in grocery stores, it is possible that Non-Hispanic Black adults have greater trust in food advertising because of the increased environmental exposure to advertising through various channels of communication. Numerous studies have demonstrated that exposure to food advertisements is linked to consumption of the foods found in those advertisements. Our results provide some initial empirical support for the cognitive mechanisms of how exposure to food advertising may contribute to consumption. Developing advertising literacy interventions to inoculate against the cognitive impacts of food advertising may be a viable strategy to limiting consumption of EDNP foods.

## Introduction

Obesity rates continue to rise among the U.S. population. The most recent data from the CDC show that more than one-third (41.9%) of U.S. adults aged 20 and older, and 19.7% of children and adolescents aged 2–19 years were obese (1, 2). Overweight and obesity are associated with a number of adverse physical and mental health outcomes, and obesity has been cited as a contributing factor to approximately 500,000 deaths in the United States per year (3).

These rising rates have led to a fascination with exploring and understanding social and environmental influences on dietary behavior. One area that has received a considerable amount of attention is the influence of food advertising. This literature has mainly explored the content of food advertising to children, the effects of that marketing, and behaviors associated with that marketing (4–6). While children and adolescents may be particularly vulnerable to the appeals of advertising, adults may also be susceptible to food advertising and it may also affect their dietary behaviors (7). In fact, 64 % (64%) of adults are exposed to television ads daily (8). Prior research has also shown that exposure to food advertisements have impacted the consumption behaviors of adults [e.g., (9, 10)]. While studies have shown an association between exposure to food advertisements and consumption behaviors, there remains a large gap in the literature in terms of understanding the cognitive mechanisms that may underlie those behaviors, particularly among adults. Currently, no singular study shows a direct relationship linking exposure to advertisements, more positive perceptions toward food advertising, and consumption of unhealthy foods among adults, though the literature suggests there may be a connection. Thus, the purpose of this study is to explore the associations between cognitions, such as attitudes and trust, toward advertising and the consumption of energy-dense, nutrient poor (EDNP) foods among adults using data from a national sample. The relationship between *perceptions* of and *trust* in food advertising and dietary behaviors has been demonstrated among adolescents (11) but has not been systematically studied among US adults. Understanding this relationship may provide insights into developing more effective health promotion strategies in support of healthy eating habits among adults in a communication environment oversaturated by food advertising.

## Literature review

While extant literature has focused on the relationship between food advertising exposure and EDNP foods consumption among adolescents, literature on the impact of food advertising on adults is limited. This small body of work has explored the amount of exposure to advertisements, the content of advertisements, the impact of exposure to specific advertisements on perceptions of those advertisements and products advertised, the impact of exposure on perceptions of advertising, and the impact of exposure on purchase intentions and consumption.

### Food advertising: content and audiences

Although adults are generally not the target subjects for research regarding their exposure to advertisements, they are still targets of advertising. In 2020, $89.8B was spent on non-digital advertisements in the United States, while $152.2B was spent on digital ads in the same market (12). Beyond adults’ exposure to advertisements in general, adults who are parents specifically are subjected to unhealthy food advertisements in particular. In a content analysis of 19,000+ advertisements from two prominent U.S. parenting magazines, 32.5% of the advertisements were for baked goods, sweets, and snacks, and 64.6% of the cereal ads were for low-nutrition cereals (13).

#### Population subgroup differences

When considering the frequency of exposure to food advertising among adults, the amount of exposure is not equal when comparing White populations and different racial, ethnic, and socioeconomic (SES) groups. Differences in exposure are tied to geographic location, and both non-Hispanic Black and Hispanic groups in the United States are disproportionately targeted. In a study of the prevalence of outdoor advertisements in a variety of metropolitan neighborhoods in the United States, results showed that Black neighborhoods had the highest density of food-related ads, Hispanic neighborhoods had a slightly lower density, and white neighborhoods had the lowest density (14). This prevalence of outdoor advertisements in racially and ethnically centralized neighborhoods suggests that billboard advertising may influence positive attitudes toward these food advertisements because attitudes toward billboards are generally more positive than negative (15). A 2021 study found that from 2007 to 2013, Non-White adults were exposed to 11% more regular soda advertisements compared to White adults (16).

Additionally, the duality of being a person from a minority race/ethnicity in a low-income neighborhood greatly increases the likelihood of exposure to food advertisements compared to high-income White neighborhoods. Researchers linked census data from specific geographic locations regarding soda consumption and BMI to the types of outdoor advertisements available in those specific neighborhoods and found that those who live in areas where there are more food advertisements have a greater chance of being overweight or obese: “for every 10% increase in food advertisements, the odds of being obese increased by 5%” (17). The prevalence of advertised unhealthy food options is not unique to the frequency of outdoor advertisements in low-SES and racial and ethnic minority neighborhoods; low-income and racial/ethnic-minority neighborhoods generally have more convenience stores and grocery stores, which offer fewer healthy food options, compared to high-income, White neighborhoods, which have more chain and non-chain supermarkets, which usually offer a larger variety in healthy food options and tend to have services such as a butcher, a deli, etc. (18).

#### Differences for Hispanic populations

The Hispanic population remains a segment of the population vulnerable to the consumption of EDNP foods because they tend to have less nutrition knowledge compared to non-Hispanic whites in the United States (19). In a television food advertising study, the near entirety (87.3%) of restaurant advertisements shown on Hispanic television channels were for fast food establishments (20). Additionally, one-quarter (24.9%) of food advertisements shown to the Hispanic market contained health claims and 58.2% of these claimed that the food being advertised was “good for one’s health,” whereas only 7.2% of food advertisements shown to the general market contained such health claims (20). Furthermore, based on the 2020 US Census data, Hispanic neighborhoods in high poverty census tracts have around half (0.55) as many supermarkets compared to Non-Hispanic White neighborhoods (21).

#### Differences for black populations

In a content analysis comparing food advertisements shown on mainstream television networks and African American television channels, 33% commercials aired were for fast food restaurants (22). Additionally, among the 553 television food advertisements shown during prime-time slots, there were more unhealthy food advertisements (for candy, fast food, soda, etc.) shown during African American programs compared to those shown during programs for the general market (22). There were also more fat-content claims made in the advertisements during the African American programs compared to the other prime-time programs (22). Recently, a study using 2020 US Census data determined that Non-Hispanic Black neighborhoods in high poverty census tracts have approximately one-third (0.38) as many supermarkets compared to Non-Hispanic White neighborhoods (21).

Given that minority populations have greater exposure to EDNP food advertising, we hypothesize that:

*H1a*: Hispanic adults will have more positive perceptions of food advertising than Non-Hispanic White adults.

*H1b*: Hispanic adults will have greater trust in food advertising than Non-Hispanic White adults.

*H1c*: Non-Hispanic Black adults will have more positive perceptions of food advertising than Non-Hispanic White adults.

*H1d*: Non-Hispanic Black adults will have higher trust in food advertising than Non-Hispanic White adults.

### Effects of advertising

If advertisements are geared toward consumers in a way that makes them feel something strongly (whether positive or negative), it will likely not only impact their attitude toward the advertisement but also their attitude toward the brand (23). Additionally, adults’ attitudes toward advertisements are generally more positive than negative. In a survey of 1,004 participants, it was found that over three quarters of respondents either had positive or neutral opinions on advertisements in general (24). Despite having generally positive attitudes toward advertising, adults have a lack of trust in ads but still disclose higher certainty in ad claims when considering their own purchase choices (24). Further, not only are adults impacted by advertisements that are intended for their age group, but they are also just as susceptible to online advertisements meant for children. Cornish (25) conducted a qualitative study in which 42 parents with children aged 5–12 were interviewed about their children’s online habits in relation to ad exposure and found that parents perceive advergames, pop-up advertisements for a product with a game incorporated, as though they are not advertisements and thus, cannot be treated the same as standard pop-up online ads.

#### Impact of food advertising

Previous research that concentrates on how food advertisements impact adults primarily focuses on whether they choose to consume specific food products as a result of exposure or how their attitude about a specific ad is impacted following exposure. A study in which 51 young women were exposed to varying numbers of soda commercials, based on their experimental condition (the women were instructed to help themselves to a variety of sodas and water bottles on the table next to them) found that the women exposed to soda commercials drank on average 1.3 ounces more soda compared to those who watched bottled water commercials (10). A related study was conducted with men and women and snack food commercials (9). The researchers found that women ate 65.7 grams more food when exposed to food commercials compared to women who were exposed to neutral commercials; however, men in the food commercial condition ate 25.5 grams more food than women in the same condition (9). Scully, Dixon, and Wakefield (26) conducted a cross-sectional phone survey in which 1,495 participants discussed their fast food eating and television watching habits. Researchers found that there was a positive association between watching television ads and consuming fast food for dinner; 55% of participants ate fast food for dinner on a biweekly or monthly basis and 22% on a weekly basis (26). McKay-Nesbitt et al. (27) conducted an experiment that exposed participants to advertisements of fictitious brands for juice, milk, and margarine. The advertisements were manipulated to have a positive-emotional appeal, a negative-emotional appeal, and a rational appeal. Older adults had a more positive attitude toward the ads with a rational appeal rather than those with a negative-emotional appeal (27).

Recent research on the impacts of food advertising on adults remains sparse, with work continuing to focus on adolescents. For example, a 2020 study found that adolescents were more responsive and attentive to unhealthy food social media advertisements (28). Not only was the response to unhealthy food advertising on social media significantly more positive than healthy and non-food advertisements, Murphy et al.’s findings also suggest that adolescents both interacted with unhealthy food advertising posts for longer and recalled the contents of the unhealthy food advertisements more frequently than healthy and non-food advertisements (28). Ultimately there has not been much work in this area focused on adult participants; with most research generally focused on whether or not consumers will eat specific foods as a result of viewing and/or being exposed to food advertisements.

#### Advertising exposure effects: positive perceptions

While many studies have demonstrated a relationship between exposure to advertisements and preference for or consumption of the foods advertised, the cognitive mechanisms underlying this relationship remain underexplored. One reason why increased and continuous consumer exposure to advertisements leads to consumption derives from the notion that higher exposure leads to increased positive consumer perceptions of the advertised product. Companies produce advertisements with the intention of enhancing their brand image through the development of positive, applicable, and reliable connections that are linked to the brand in the consumer’s subconscious (29, 30). Once the company establishes brand familiarity with a consumer, repeated exposures will result in the consumer subconsciously making associations with additional exposures thereafter, enforcing a consumer’s brand loyalty and thus urging the consumer to purchase products from the advertised brand (6, 30). This process also works with food advertisements: repeated and persistent advertisement exposure from the same food brands leads to positive associations of the brand for the consumer, and social media and digital advertisement interactivity positively influences brand engagement (31). This, in turn, likely leads to both the purchasing and consumption of the product, further reinforcing the consumer’s brand devotion (31, 32).

#### Developing trust

Consumers who are repeatedly exposed to certain brands will undoubtedly have greater brand recognition of them. Another outcome of repeated exposures is that consumers might also develop trust in the brand, which should also result in greater purchasing intentions and behaviors (30). Whether or not a consumer generally trusts advertising can impact how they interact with the brand; if they lack trust in advertising, they are less likely to develop trust in specific advertisements (33). Current literature regarding consumers’ trust in advertisements is limited (34–37); however, a related area of research is advertising skepticism, which can be characterized as a consumer rejecting the content of advertisements (38). Whether or not consumers are skeptical about advertising derives from how the consumer interacts with advertising. This may include the cognitions they engage when they process advertisements, how they depend upon information within advertisements, how they develop brand assumptions, how they respond to advertising strategies, and intent to purchase (39). As well, this may also include their perceptions of the advertisement itself [i.e., how much they like the advertisement, who they believe the advertisement is targeted toward, how appropriate they think the message or product advertised is (40)]. Additionally, the growing prevalence of digital and social media marketing presents another determinant in developing trust in food advertising: the presence of role models and influencers. The presence of influencers and role models in digital and social media food advertising may influence brand perception and thus positively impact consumer trust (41). The degree to which a consumer trusts advertisements can impact a consumer’s intentions and attitudes toward food, which makes it a concept worth considering in relation to food advertising.

There has also not been much work in the impacts of food advertising exposure effects on adults, with most recent work focused on the effects on children and adolescents such as Backholer et al. (42) research on the impacts of differential unhealthy food advertising exposure by SEP and race/ethnicity among children, and Boyland et al’s (43) work on the influence of food and nonalcoholic beverage advertising on the health and dietary habits of children and adolescents.

Given that the purpose of advertising is to generate positive perceptions and greater trust, and positive perceptions and trust should lead to greater consumption,

*H2*: We hypothesize that more (a) positive perceptions of and (b) higher trust in food advertising will be positively associated with more frequent consumption of EDNP foods and drinks among adults.

## Methods

### Research design and nature of study

The current study is a secondary data analysis of publicly available data. Data for the current study was extracted from the US National Cancer Institute’s Family Life, Activity, Sun, Health and Eating (FLASHE) Survey, which is a cross-sectional, web-based panel survey of adolescent-parent dyads.^1^ The FLASHE survey was designed to assess psychosocial, generational (parent-adolescent), and environmental predictors of cancer-preventive behaviors and includes measures on lifestyle behaviors such as diet and physical activity, sleep, sun-safety, and tobacco use. Data was collected using two separate web-based surveys, with one focusing on diet and the other focusing on physical activity, administered between April–October 2014 to dyads of parents and their adolescent children (ages 12–17), who were the target population for the overall FLASHE project. Detailed data collection methods, such as sampling techniques, data collection processes and procedures, are described elsewhere (44). The institutional review boards of the NCI and Westat approved the study in 2014.

### Unit of analysis and sample size

A total of 1,945 dyads enrolled in the study, resulting in a 38.7% response rate. While dyadic data are available from the FLASHE data set, in this study, we extracted the data from the adults only, as adults are the target population for our hypotheses. A total of 1,745 adults completed the diet surveys (89.7% response rate). Complete case analysis with listwise deletion was applied, resulting in a final analytical sample of 1,535 adults. Respondents were excluded if data for any of the variables included in the analytical models were not available for them. Since we conducted a secondary data analysis study, we used all data available and did not calculate a sample size. Our final analytic sample of 1,535 is a sufficient sample to detect small effects at *p* ≤ 0.05.

### Measures

#### Independent variable: perceptions of food advertising

Two items were developed and cognitively tested for FLASHE to measure perceptions of food advertising. Survey respondents were asked to respond to the following: “Please think about messages you see or hear on television, magazines, radio, internet or billboards about foods and drinks. Please select how much you disagree or agree with each of the statements below. (1) When I see advertisements for foods or drinks, I want to try the advertised foods or drinks. (2) When I see advertisements for foods or drinks, I think the advertised foods or drinks will taste good.” Response options were based on a five-point Likert-type scale and included: strongly disagree (1), somewhat disagree (2), neither disagree nor agree (3), somewhat agree (4), strongly agree (5). Higher scores indicated more positive perceptions of food advertising. A mean score of these two items was calculated to create a new variable to capture perceptions toward advertising (Cronbach’s alpha = 0.75). This variable was operationalized as a proxy for general perceptions of food advertising.

#### Independent variable: trust in food advertising

Independent variable: trust in food advertising was assessed through one item. Respondents were asked to respond to the following question: “Please think about messages you see or hear on television, magazines, radio, internet or billboards about foods and drinks. Please select how much you disagree or agree with each of the statements below: When I see advertisements for foods or drinks, I trust the messages advertised.” Response options were based on a five-point Likert-type scale and included: strongly disagree (1), somewhat disagree (2), neither disagree nor agree (3), somewhat agree (4), strongly agree (5), with higher scores indicating greater trust.

#### Dependent variable: daily frequency of consumption of EDNP foods and drinks

A daily frequency consumption variable was computed where the nine food products included were summed, including soda, sweetened fruit drinks, sports drinks, candy, chocolate, cookies, cake, potato chips, and fried potatoes. Items for each of the foods were presented as follows: “During the past 7 days, how many times did you eat [food product]?” Response options included: I did not eat [food product] in the past 7 days; 1–3 times in the past 7 days; 4–6 times in the past 7 days; 1 time per day; 2 times per day; 3 or more times per day. The items were summed based on a procedure described in more detail previously (11, 45, 46).

#### Sociodemographic variables

Sociodemographic variables included sex, age, race/ethnicity, education level (used as a proxy for socio-economic status), and weight status. Each variable was categorized as follows: sex (male, female); age (continuous); race/ethnicity (Non-Hispanic White; Non-Hispanic Black; Hispanic; Other Race); education (less than high school; high school degree; some college; college graduate or more); and self-reported BMI, which was categorized into standard weight status based on the following criteria: underweight, Below 18.5 BMI; healthy or normal weight, 18.5–24.9 BMI; overweight, 25.0–29.9 BMI; obese, 30.0 and above BMI (47).

### Data analysis tools and techniques

We used SAS 9.3 to conduct all statistical analyses. First, we generated descriptive statistics for all demographic variables. To assess differences between demographic subgroups for advertising perceptions and trust, one-way ANOVAs with planned contrasts were conducted. We used bivariate linear regression models to examine the associations between (1) perceptions of advertising and consumption of EDNP foods and drinks; and (2) trust in food advertising and consumption of EDNP foods and drinks. Then, we used multivariable regression models to examine these relationships while controlling for socio-demographic factors. The threshold for statistical significance was set at *p* ≤ 0.05.

## Results

### Descriptive statistics

The majority of the sample was female (73.49%) and/or non-Hispanic White (70.36%; see Table 1). Almost two thirds of the sample was either overweight or obese (31.21% overweight and 30.88% obese). Approximately half of the sample earned a 4-year college degree or higher (47.04%). The age range was moderately wide (20–73), but given the spread of the range, the majority of the sample was concentrated in a relatively narrow window with the mean age being 43.88 (SD = 7.70). Participants’ perceptions toward food advertising was slightly more positive. On a 1–5 scale with 5 being a more positive perception, the mean response was 3.31 (SD = 0.94). Participants’ trust in food advertising was almost neutral. On a 1–5 scale with 5 being a greater trust, the mean response was 2.60 (SD = 1.03). The mean daily frequency of consumption of the nine EDNP foods and drinks (soda, sweetened fruit drinks, sports drinks, candy, chocolate, cookies, cake, potato chips, and fried potatoes) was 2.07 times per day (SD = 1.62).

**Table 1:** Descriptive statistics of adults (*n* = 1,535).

Sex	
Male Female	26.51% 73.49%
Race/Ethnicity	
Non-Hispanic White Hispanic Non-Hispanic Black Other	70.36% 7.62% 18.31% 3.71%
Weight status	
Underweight Healthy weight Overweight Obese	1.30% 36.61% 31.21% 30.88%
Education	
Less than a high school degree A high school degree or GED Some college but not a college degree 4 year college degree or higher	1.24% 16.48% 35.24% 47.04%
Age	Mean = 43.88 SD = 7.70 Range = 20–73
Perceptions toward food advertising Mean score calculated from two items Scale, 1–5, 5 = more positive perceptions	Mean = 3.31 SD = 0.94 Range = 1–5
Trust in food advertising Scale, 1–5, 5 = greater trust	Mean = 2.60 SD = 1.03 Range = 1–5
Daily frequency of consumption of EDNP foods and drinks Combined intake calculated for 9 items: soda, sweetened fruit drinks, sports drinks, candy, chocolate, cookies, cake, potato chips, and fried potatoes	Mean = 2.07 times per day SD = 1.62 Range = 0–8

### Sociodemographic differences in perceptions toward and trust in food advertising

One way independent ANOVAs were conducted to assess differences between population subgroups on the two key dependent variables of interest, perceptions toward food advertising and trust in food advertising. There were significant differences between race/ethnicity, [*F*(3, 1,531) = 8.59, *p* < 0.0001, *η* = 0.02, and education level, *F*(3, 1,531) = 3.27, *p* = 0.02, η = 0.01] for perceptions toward food advertising (see Table 2). There were also significant differences for these two variables for trust in food advertising [race/ethnicity, *F*(3, 1,531) = 8.69, *p* < 0.0001, η = 0.02; education level, *F*(3, 1,531) = 5.24, *p* = 0.01, η = 0.01]. There were no significant differences in perceptions toward food advertising or trust in food advertising based on age or sex.

**Table 2:** One-way ANOVA of perceptions toward food advertising and trust in food advertising.

	Perceptions toward food advertising				Trust in food advertising			
	df	F	*η*	*p*	df	F	*η*	*p*
Sex	1	1.44	0.00	0.23	1	0.17	0.00	0.68
Age	5	0.94	0.058	0.45	5	1.43	0.071	0.21
Race/Ethnicity	3	8.59	0.02	**<0.0001**	3	8.69	0.02	**<0.0001**
Education level	3	3.27	0.01	**0.02**	3	5.24	0.01	**<0.01**

Bolded values indicate statistical significance at *p* < 0.05.

**Figure 1:**
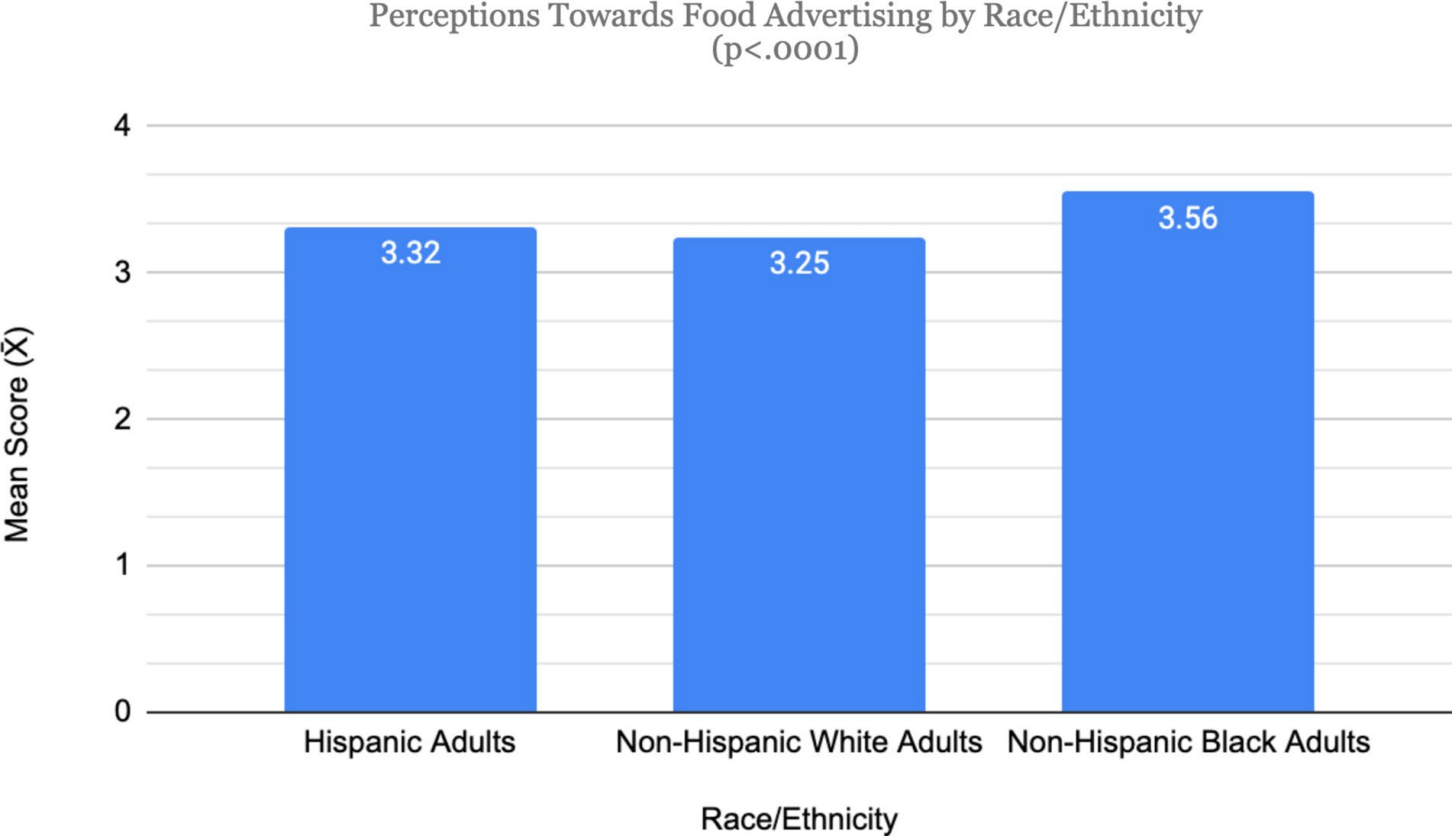
Perceptions toward food advertising by racial/ethnic groups.

**Figure 2:**
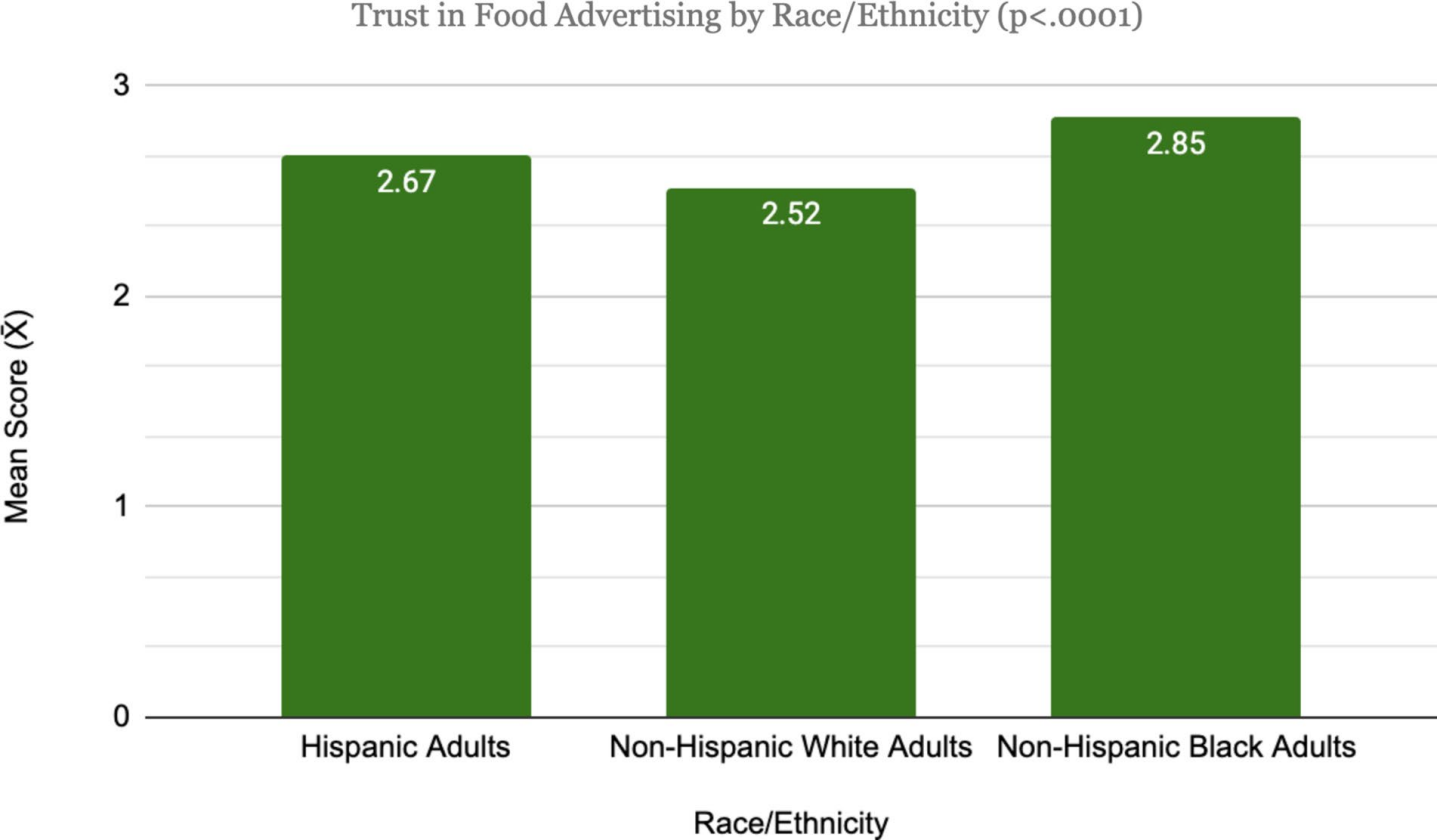
Trust in food advertising by racial/ethnic groups.

Planned contrasts revealed a statistically significant difference in perceptions toward advertising between Hispanic adults (mean = 3.32) and Non-Hispanic Whites (mean = 3.25; *t*[1531] = 2.31, *p* = 0.02); though the difference is negligible so H1a was not supported. There was no significant difference between Non-Hispanic Whites and Hispanics (mean = 2.67) on the level of trust in food advertising (*t*[1531] = 0.34, *p* = 0.734); thus, H1b was not supported. Differences in perceptions toward advertising between Non-Hispanic Whites and Non-Hispanic Blacks (mean = 3.56) were not significant, *t*(1531) = −0.02, *p* = 0.98; thus, H1c was not supported. For differences in trust in food advertising, planned contrasts show that there was a statistically significant and meaningful difference, *t*(1531) = −2.01, *p* = 0.04, between Non-Hispanic Blacks (mean = 2.85) and Non-Hispanic Whites (mean = 2.52); thus, H1d was supported (Figures 1, 2).

### Perceptions toward food advertising and consumption of EDNP foods

There are consistent positive relationships between perceptions of and trust in food advertising and the daily frequency of consumption of EDNP foods and drinks among adults as shown in the unadjusted and adjusted models. The unadjusted bivariate model with perceptions of food advertising as the predictor and daily frequency of consumption of EDNP foods and drinks as the dependent variable (table not shown) shows that as perceptions toward food advertising become more positive, consumption increases (*b* = 0.25, *p* < 0.0001). Similarly, the bivariate unadjusted model with trust in food advertising as the predictor and consumption of EDNP foods and drinks as the outcome shows a positive relationship (*b* = 0.25, *p* < 0.0001; table not shown). In the multivariable unadjusted model with both predictors and daily frequency of consumption as the outcome, both perceptions toward food advertising and trust in food advertising are significant (Table 3). In the adjusted model, as perceptions toward food advertising increased among adults, there was an associated increase in daily frequency of consumption of EDNP foods and drinks (*b* = 0.15, *p* < 0.0001). The same relationship was observed for trust in food advertising: the more adults agreed that they trusted food advertising, the higher the associated increase in daily frequency of EDNP food and drink consumption (*b* = 0.13, *p* < 0.0001). Older adults and Non-Hispanic Black adults had a higher daily frequency of consumption of EDNP foods and drinks compared to younger adults and Non-Hispanic White adults, respectively. The model was able to explain 12% of the variance with an R-square value of 0.12. H2 was thus supported.

**Table 3:** Association between perceptions toward and trust in food advertising and daily frequency of consumption of EDNP foods and drinks (*n* = 1,535).

	Multivariable unadjusted					Multivariable adjusted				
	*b*	*Β*	SE(B)	*t*	*p*	*b*	*Β*	SE(B)	*t*	*p*
Perceptions toward food advertising	0.29	0.17	0.04	5.85	**<0.0001**	0.27	0.15	0.04	5.53	**<0.0001**
Trust in food advertising	0.24	0.16	0.05	4.74	**<0.0001**	0.21	0.13	0.05	4.16	**<0.0001**
Sex										
Male						0.48	0.13	0.09	5.17	**<0.0001**
Female (ref)						–	–	–	–	–
Age						−0.02	−0.09	0.005	−3.83	**0.0001**
Race/ethnicity										
Non-Hispanic White (ref)						–	–	–	–	–
Hispanic						0.35	0.06	0.19	1.87	0.06
Non-Hispanic Black						0.25	0.06	0.11	2.22	**0.02**
Non-Hispanic other						−0.33	−0.03	0.18	−1.84	0.06
Education level										
Less than HS (ref)						–	–	–	–	–
HS or GED						−0.40	−0.09	0.40	−1.00	0.31
Some college, no degree						−0.53	−0.16	0.39	−1.35	0.17
4-year college degree or higher						−0.55	−0.17	0.39	−1.41	0.15
Weight status										
Healthy weight (ref)						–	–	–	–	–
Underweight						0.64	0.04	0.45	1.41	0.15
Overweight						0.06	0.02	0.09	0.74	0.45
Obese						0.14	0.04	0.09	1.43	0.15

Bolded values indicate statistical significance at *p* < 0.05.

## Discussion

This study is one of the first studies to explore the relationship between *adult*s’ perceptions toward and trust in food advertising and the consumption of EDNP foods and drinks. Results from the current study indicate that US adults generally have positive perceptions of food advertising and trust in food advertising. These perceptions mirror those from the study of adolescents, who also had positive perceptions of and trust in food advertising (11).

The results from this study show that there were differences between Non-Hispanic Black adults and Non-Hispanic White adults regarding level of trust in food advertising. Non-Hispanic Black adults had the greatest trust in food advertising and their level of trust in food advertising was significantly different than the level of trust among non-Hispanic White adults. This finding is supported by Cervi et al.’s (48) conclusion that susceptibility to SSB advertisements was more prominent among Non-Hispanic black youths compared to their non-Hispanic white counterparts, and Fareed et al.’s (49) determination that the increased levels of trust in health advertising shown by non-Hispanic black and Hispanic adults compared to non-Hispanic whites could be attributed to differences in their access to information sources and informal community sources. Given that previous studies have shown that ethnic minority groups, particularly Non-Hispanic Blacks, are more likely to be exposed to more TV food advertisements (16, 22), billboards (14, 17), and grocery stores (21) for less healthful foods (e.g., EDNP foods and drinks including, fast food, candy, soda), it is possible that Non-Hispanic Black adults have greater trust in food advertising because of the increased environmental exposure to advertising through various channels of communication.

Perhaps the most interesting finding is that adults who have more favorable perceptions of and higher trust in food advertising show higher frequency of consumption of EDNP foods and drinks compared to those who had less positive perceptions and less trust toward food advertising. Numerous studies have demonstrated that exposure to food advertisements is linked to consumption of the foods found in those advertisements (4–6, 43, 50–52). The present study further illuminates this research by providing some initial empirical support as to *how* exposure to food advertising may contribute to consumption through cognitive mechanisms.

Health behavior change theories that are often used to design public health interventions to influence dietary behaviors also include cognitive constructs such as attitudes and expectations (e.g., Theory of Planned Behavior, Social Cognitive Theory), highlighting the importance of developing positive attitudes, or perceptions, toward a given behavior. Attitudes have been defined in a number of ways [see (53)], with Eagly and Chaiken (54) offering a widely accepted one: “a psychological tendency that is expressed by evaluating a particular entity with some degree of favor or disfavor.” In the design of health behavior interventions using behavior change theories, there is a system of constructs that scholars and practitioners will use to develop intervention components with the intent of shifting cognitions related to these constructs. For example, in Theory of Planned Behavior, attitudes, subjective norms, and perceived behavior control are assumed to influence behavioral intent that, in turn, influences behavior, with intention as the immediate antecedent of behavior (55, 56). However, in advertising, it is unclear whether these other constructs are considered in the design and development of campaigns.

Since advertising seemingly has the sole goal of generating positive cognitions, such as attitudes and trust, about the products and brands advertised, which then may lead to purchase intentions and consumption behaviors (31, 32, 41, 57), further exploration of cognitive responses to food advertising and how to inoculate against them could be one fruitful way to mitigate the effects of food advertising on the consumption of EDNP foods rather than using traditional health behavior change interventions. One potential strategy to achieve this is through the development and implementation of media and advertising literacy interventions (11, 58, 59).

While much scholarly attention has been paid to media literacy and the many ways to define and approach media literacy interventions (60, 61), media and advertising literacy interventions are generally constructed and integrated in a manner that helps individuals cultivate critical thinking skills that revises the way they access and process media and advertising messages (60, 62, 63). These interventions have shown to be effective in modifying the development of cognitive associations and attitudes, especially that of advertisements and advertised products as a way to impact the consumption of those products; however, most studies have been focused on children and adolescents and on topics such as alcohol and tobacco (41, 64–66).

Media literacy interventions focused on diet and nutrition however, have been limited and have thus far only shown modest effects on dietary behaviors (67), with only two studies focused on adults, one of which focused solely on sugar-sweetened beverages (68, 69). Evans et al. (67) employed a media literacy intervention in an attempt to increase fruit and vegetable consumption among fourth-and fifth-grade children through changing their home food environment. The curriculum revolved around the children creating campaigns to encourage their parents to buy healthier foods. The children who participated in the program learned how to create a logo, a slogan, key messages to encourage their parents to have more fruits and vegetables in the home, and multiple media products to deliver the messages to the parents. A total of 39 students participated in the study, with 18 in the intervention group and 21 in the control group. When compared to the control group, children in the intervention group reported greater motivation to eat fruits and vegetables, increased fruit and vegetable accessibility at home, and increased parental social support with regard to fruit and vegetable consumption. However, the intervention was not effective in changing fruit and vegetable consumption.

Meanwhile, Hindin et al. (69) designed a four-week media literacy nutrition education curriculum in which they recruited 35 parents of children participating in Head Start programs. Topics included understanding children’s perceptions of advertising, analyzing and talking to children about television commercials for food products, identifying truth in advertising, responding to children’s requests for advertised food products, and reading food labels. A comparison of pre-and post-test measures revealed that the curriculum had significant effects on parents’ understanding of and attitudes toward television food commercials, TV mediation behaviors, understanding of and ability to read food labels, and outcome expectations related to talking about food commercials with children and responding to children’s requests for advertised foods at the grocery store. Considering these findings, the authors suggested that media literacy is an effective approach to teach parents how to critically analyze various forms of food-related media content.

Finally, Chen et al. (68) recruited 296 adults over 18 years with various health literacy levels to partake in a controlled six-month intervention regarding sugar-sweetened beverage advertising that involved media literacy lessons and interactive voice response phone calls. Topics included health literacy concepts such as media, print, and oral literacy, cultural literacy, as well as numeracy. A comparison of baseline and post-program assessments revealed that media literacy education was an effective intervention in increasing sugar-sweetened beverage advertising related media literacy skill sets such as critical examinations of food media, identifying health-related omissions, and identifying persuasive techniques utilized in food advertising and marketing among adult participants. Based on the findings of the intervention, the authors concluded that media literacy education is a crucial intervention that can be utilized for all adults, regardless of the participant’s health literacy level. With only a handful of studies to date, the development of media literacy interventions to alter cognitions and perceptions of food advertising to impact dietary behaviors merits further study, among adolescents and adults alike.

Public policy reforms could be another viable strategy to mitigate the effects of advertising on dietary behaviors. Given that ethnic minority groups are exposed to more advertisements for unhealthy foods, limiting food advertising to these groups could be one potential strategy. This could be achieved by limiting advertisement placements for certain media outlets that are known to have audiences from minority groups. Another strategy could be the taxation of unhealthy foods, such as sugar-sweetened beverages (SSBs). While this would not be a strategy directly targeting advertising, it is certainly an effective way to prevent consumption of EDNP foods. A recent systematic review and meta-analysis of 62 studies on the effects of SSB taxes showed that SSB taxes were associated with higher prices and lower sales of taxed beverages (70).

## Limitations

There are several limitations to this study. First, while these data are drawn from a national panel, they are not weighted to be nationally representative. Second, the national data utilized in this study is from 2014 and is not the most current. The digital advertising landscape has changed significantly since then, due to the introduction of social media platforms where people are exposed to food advertising through targeted ads and influencers; thus, these results may not be as generalizable to the current media landscape. Even so, the majority of this work is focused on the effects of social media advertising on children and adolescents, and not on adults (41, 43, 71). Third, given that the data are cross-sectional, temporality and causal inference cannot be concluded (72). Fourth, the data are self-reported and are subject to social desirability bias (73, 74). Additionally, the effect sizes are generally small, but are not surprising given the use of national data (75). Regarding measures included in the study, dietary screeners used to assess ENDP food and drink intake may not provide the most precision for assessing EDNP food and beverage intake, though they are practical for large studies (76). The study included two measures on perceptions toward food advertisements and one item on trust in food advertisements and did not assess actual exposure to food advertisements.

While this study had limitations, it also had many strengths. One strength is that it is one of the first studies to examine trust in and perceptions of food advertising as variables to further aid our understanding of their association with the consumption of EDNP foods and drinks among adults in the US. Such a large sample of data on variables related to adults’ perceptions of and trust in food advertising has not been previously available; therefore this study exhibits high external validity. Our findings contribute to our understanding of potential cognitive mechanisms underlying the association between advertising exposure and consumption. Given that recent research on food advertising, including exposure to and impacts of, among adults remains sparse, we believe more research is needed in this domain.

## Conclusion

As one of the first studies to investigate the relationship between perceptions toward and trust in food advertising among adults using a national sample, our findings show that trust and perceptions toward food advertising were positively and significantly associated with the daily consumption of EDNP foods and drinks among US adults. Understanding this relationship may provide insights into developing more effective health promotion strategies in support of healthy eating habits in a communication environment oversaturated by food advertising. While many factors may influence the complex behavior of dietary choice, our study provides initial evidence for a potential cognitive mechanism influenced by food advertising and marketing. The findings in the present study suggest that employing media and advertising literacy interventions that alter cognitive responses to food advertising may be a worthwhile strategy in efforts to reduce the impact of food marketing and the consumption of EDNP foods and drinks among both adolescents and adults.

## Data Availability

The datasets presented in this study can be found in online repositories. The names of the repository/repositories and accession number(s) can be found at: https://cancercontrol.cancer.gov/brp/hbrb/flashe-study/flashe-terms?destination=/brp/hbrb/flashe-study/flashe-files.

## Footnotes

^1^ https://cancercontrol.cancer.gov/brp/hbrb/flashe.html
